# Identification of a Retroelement-Containing Human Transcript Induced in the Nucleus by Vaccination

**DOI:** 10.3390/ijms20122875

**Published:** 2019-06-13

**Authors:** Tomoyuki Honda, Keiko Takemoto, Keiji Ueda

**Affiliations:** 1 Division of Virology, Department of Microbiology and Immunology, Osaka University Graduate School of Medicine, Osaka 565-0871, Japan; kueda@virus.med.osaka-u.ac.jp; 2 Institute for Frontier Life and Medical Sciences, Kyoto University, Kyoto 606-8397, Japan; ktakemot@infront.kyoto-u.ac.jp

**Keywords:** endogenous retrovirus, innate immunity, interferon, MLT-int, retroelement, vaccination

## Abstract

Endogenous retroelements constitute almost half of the mammalian genome. Given that more than 60% of human genomic bases are transcribed, transcripts containing these retroelements may impact various biological processes. However, the physiological roles of most retroelement-containing transcripts are yet to be revealed. Here, we profiled the expression of retroelement-containing human transcripts during vaccination and found that vaccination upregulated transcripts containing only particular retroelements, such as the MLT-int element of endogenous retroviruses. MLT-int-containing transcripts were distributed mainly in the nucleus, suggesting their unique roles in the nucleus. Furthermore, we demonstrated that MLT-int RNA suppressed interferon promoter activity in the absence of immune stimuli. Based on these lines of evidence, we speculate a model of a role of the previously unnoticed MLT-int element in preventing excess innate immune activation after elimination of immune stimuli. Our results may emphasize the importance of retroelement-containing transcripts in maintaining host immune homeostasis.

## 1. Introduction

Endogenous retroelements constitute almost half of the mammalian genome [1]. Among them, endogenous retroviruses (ERVs) are fossil records of ancient retroviruses and occupy ~10% of the human genome [1]. Although some ERVs still possess open reading frames encoding functional proteins [2], ERV replication is yet to be demonstrated in humans [3]. Considering that more than 60% of the human genome is transcribed [4], ERV-containing transcripts, regardless of their coding potential, make up a substantial fraction of transcriptome and form the “endogenous retrovirome”. The endogenous retrovirome may exert biological functions, similarly to the gut microbiome; however, the biological significance of most ERV-containing transcripts remains unclear. 

Recent advances in transcriptional profiling accelerated our understanding of the expression landscapes of the endogenous retrovirome in various biological conditions [5] and provided clues regarding how ERVs function. For example, a small number of distinct ERVs are strongly induced during B-cell activation, while ERVs are widely induced during B-cell transformation [5]. These observations suggest that particular ERVs or ERV-containing transcripts may play roles in host immune tuning, and that dysregulation of such ERV expressions may contribute to pathological conditions. However, the way how these ERV-containing transcripts exert their function is still obscure. Here, we profiled retroelement-containing human transcripts during immune stimulation and identified that the transcripts which contained a particular ERV were strongly upregulated during the stimulation. The upregulated ERV-containing transcripts were distributed mainly in the nucleus, suggesting their potential roles in the nucleus. Our finding may represent a novel mechanism through which ERVs play a role in host immune system. 

## 2. Results

### 2.1. Expression Profiling of Retroelement-Containing Transcripts in the Human Blood during Vaccination

**Figure 1:**
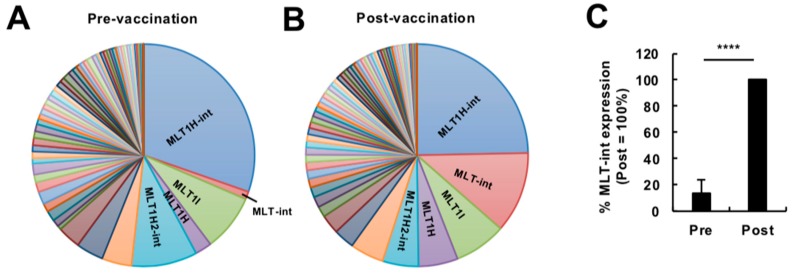
Expression profiling of transcripts containing retroelements in the human blood during vaccination at a high dose. (**A**,**B**) Diversity of transcripts containing the mammalian apparent LTR-retrotransposon (MaLR) elements expressed in peripheral blood cells before (**A**) and at 24 h after (**B**) vaccination at a high dose (GSE98212, high-dose, *N* = 10). The vaccination was administered to humans in a previous study using a replication-defective simian adenovirus expressing two *Leishmania* proteins, KMP-11 and HASPB, at a high dose (for more detail, see Reference [6]). The top five elements are indicated. (**C**) Expression of MLT-int-containing transcripts before (Pre) and after (Post) vaccination. The percentage of MLT-int expression (reads per kilobase of exon per million mapped reads, RPKM) before vaccination to that after vaccination for each subject was calculated. The values are expressed as the mean ± SD; **** *p* < 0.001.

**Figure 2:**
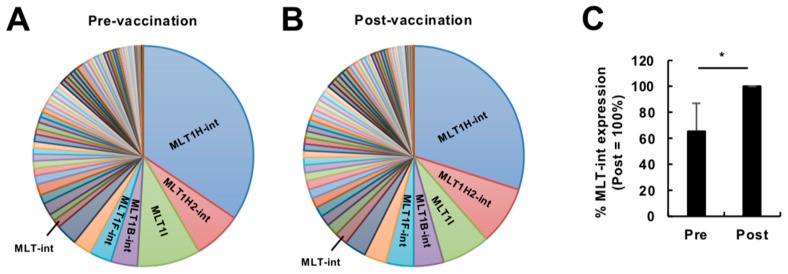
Expression profiling of transcripts containing retroelements in the human blood during vaccination at a low dose. (**A**,**B**) Diversity of transcripts containing the MaLR elements expressed in peripheral blood cells before (**A**) and at 24 h after (**B**) vaccination at a low dose (GSE98212, low-dose, *N* = 5). The vaccination was administered to humans in a previous study using a replication-defective simian adenovirus expressing two *Leishmania* proteins at a low dose (for more details, see Reference [6]). The top five elements and MLT-int are indicated. (**C**) Expression of MLT-int-containing transcripts before (Pre) and after (Post) vaccination. The percentage of MLT-int expression (RPKM) before vaccination to that after vaccination for each subject was calculated. The values are expressed as the mean ± SD; * *p* < 0.05.

To reveal how the human endogenous retrovirome is affected by immune stimulation, we analyzed publicly available RNA sequencing (RNA-seq) data (high-dose vaccination subjects in GSE98212, *N* = 10) of whole blood cells from subjects of a vaccine trial using a replication-defective simian adenovirus expressing two *Leishmania* proteins, reported previously [6]. We firstly compared the composition of retroelement-containing transcripts expressed before and at 24 h after vaccination at a high dose (Figure S1A,B). We categorized retroelements in the transcripts into six groups, i.e., mammalian apparent LTR-retrotransposon (MaLR), endogenous retrovirus 1 (ERV1), ERVK, ERVL, long interspersed element (LINE), and short interspersed element (SINE) [7]. Overall, a proportion of retroelement-containing transcripts were almost comparable between pre- and post-vaccination (Figure S1A,B). However, detailed inspection revealed that, among retroelement-containing transcripts, transcripts containing MLT-int, which belongs to the MaLR family [8], showed the most robust upregulation by vaccination (Figure 1; Figure S1C). We, therefore, focused on MLT-int-containing transcripts for further investigation. Upregulation of MLT-int RNA was strongly biased toward a sense polarity (Figure S1C). Among the MLT-int loci, an *MLT-int* element on chromosome 4, which is located within the *HERC5* gene, was robustly upregulated (Figure S1D). To confirm this observation, we analyzed a second set of RNA-seq data (low-dose vaccination subjects in GSE98212, *N* = 5), which was also reported previously [6]. We compared the compositions of all MaLR-containing transcripts expressed before and at 24 h after vaccination (Figure 2A,B). Although the overall difference was less prominent than that of the high-dose vaccination dataset, expression of MLT-int-containing transcripts was consistently upregulated at 24 h after vaccination (Figure 2A–C). These results indicate that immune stimulation by vaccination exhibits a unique induction pattern of retroelements, such as strong upregulation of the MLT-int element.

### 2.2. Localization of MLT-int-Containing Transcripts in the Nucleus

**Figure 3:**
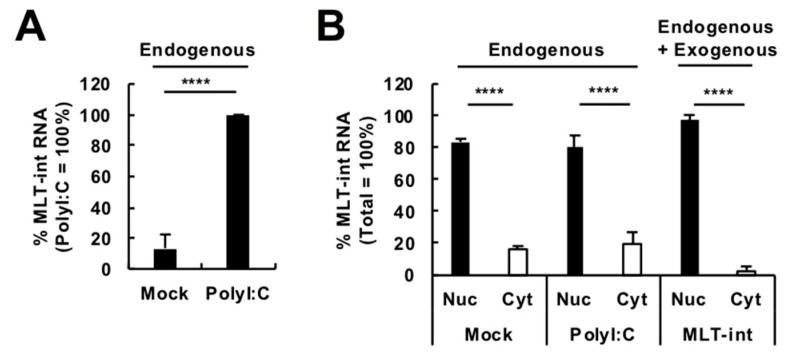
Expression of MLT-int-containing transcripts in cultured cells. (**A**) Induction of MLT-int RNA by a polyI:C treatment. (**B**) Subcellular localization of MLT-int RNA in Mock, polyI:C-treated, and MLT-int-expressing 293T cells. The values are expressed as the mean ± standard error (SE) of at least four independent experiments; **** *p* < 0.001.

We next evaluated whether innate immune stimulation can upregulate the expression of MLT-int-containing transcripts in cultured cells. We stimulated 293T cells with polyI:C for 24 h and examined the expression of MLT-int-containing transcripts. Similar to MLT-int induction after immune stimulation by vaccination (Figure 1 and Figure 2), MLT-int expression was upregulated by polyI:C treatment in 293T cells (Figure 3A). Basically, an MLT-int element is expressed using corresponding upstream long terminal repeats (LTRs). We identified the *MLT1A0* LTR sequence, which is known to accompany the MLT-int sequence [9], upstream of the *MLT-int* on chromosome 4. We subcloned the *MLT1A0* sequence and evaluated the promoter activity. Luciferase expression downstream of the *MLT1A0* sequence was almost at the background level regardless of the presence of polyI:C (Figure S2A), suggesting that the *MLT1A0* sequence did not contain sufficient promoter activity in these contexts. Because MLT-int expression was robustly upregulated by vaccination and is located within the *HERC5* gene (Figure 1C and Figure 2C; Figure S1D), which is an interferon (IFN)-stimulated gene (ISG) [10], MLT-int RNA induction is likely affected by the *HERC5* promoter activation in response to immune stimuli. Consistently, we detected robust upregulation of the HERC5 expression in the presence of polyI:C (Figure S2B). However, as mapped reads were enriched at the *MLT-int* locus after vaccination (Figure S1D), we speculated that, in addition to the *HERC5* promoter-driven MLT-int-containing transcripts, other MLT-int-containing transcripts were also upregulated by immune stimuli. We then investigated a subcellular localization of MLT-int-containing transcripts. As shown in Figure 3B, MLT-int-containing transcripts were distributed mainly in the nucleus regardless of the presence of polyI:C, while a protein-coding GAPDH messenger RNA (mRNA) was in the cytoplasm (Figure S2C). These results suggest that MLT-int-containing transcripts play a unique role in the nucleus.

### 2.3. Suppression of the IFN Promoter Activity by MLT-int in the Absence of Immune Stimuli

**Figure 4:**
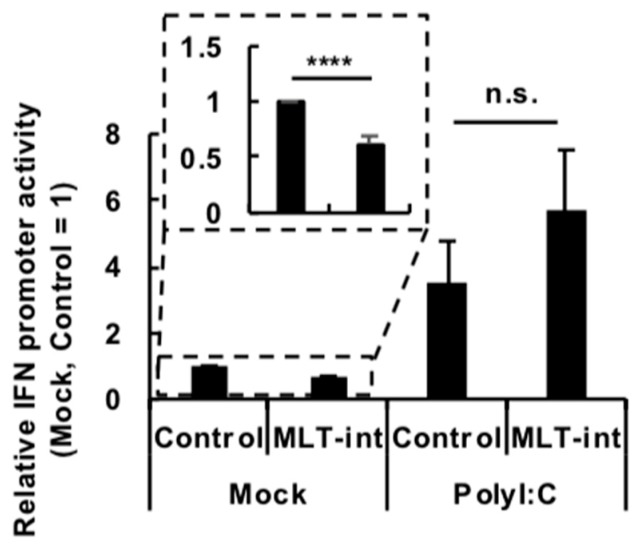
Suppression of interferon (IFN) promoter activity by MLT-int RNA in the absence of immune stimuli. The 293T cells were transfected with a plasmid expressing MLT-int RNA. After 24 h of incubation, the cells were treated with polyI:C and further incubated for 24 h. The values are expressed as the mean ± SE of at least four independent experiments; **** *p* < 0.001; n.s., no significance.

Because immune stimuli, such as vaccination or a polyI:C treatment, upregulated MLT-int-containing transcripts, we reasoned that the transcripts may have a function related to immune responses. We, therefore, tested whether MLT-int RNA plays a role in IFN signaling, i.e., IFN induction and/or responses after IFN induction. To evaluate the effect on IFN induction by MLT-int RNA expression, we conducted an IFN promoter assay using a Pol II-driven expression plasmid encoding the *MLT-int* element that contained the *MLT-int* sequence and the upstream *MLT1A0* LTR. In the absence of immune stimuli, MLT-int RNA expression suppressed the IFN promoter activity (Figure 4, “Mock”). On the other hand, when the IFN promoter was stimulated by polyI:C, MLT-int RNA expression did not affect the promoter (Figure 4, “PolyI:C”). The subcellular localization of exogenous MLT-int RNA was mainly in the nucleus, similar to that of endogenous MLT-int-containing transcripts (Figure 3B; Figure S2C). IFN stimulates the promoter activity of an IFN-stimulated response element (ISRE) to induce ISG expressions. We, therefore, evaluated responses after IFN induction in the presence or absence of MLT-int RNA by measuring the luciferase activity driven by the ISRE promoter. ISRE promoter activity in 293T cells was comparably upregulated by exogenous IFN treatment regardless of MLT-int RNA expression (Figure S3). Taken together, MLT-int RNA appears to suppress IFN induction in the absence of immune stimuli but not IFN induction during immune stimulation or responses after IFN induction. 

## 3. Discussion

In this study, we investigated the transcriptional landscapes of retroelement-containing human transcripts during immune stimulation by vaccination and revealed that vaccination did not change overall landscapes of retroelement-containing transcripts (Figure S1A,B). However, detailed inspection revealed that the expression of several retroelement-containing transcripts, such as those containing the MLT-int elements, was robustly upregulated (Figure 1 and Figure 2; Figure S1C). This robust induction of a limited number of retroelements following immune stimulation is consistent with a previous finding in the case of B-cell stimulation [5]. Induction of specific retroelements after immune stimulation prompts us to speculate the roles of these MLT-int-containing transcripts in immune tuning. 

Compared with the reported functions of retroelements, the function of MLT-int described in this report is unique. Currently, most retroelement functions are categorized into two groups, those related to reproductive processes and those related to regulation of host immunity [11]. Examples of the former functions include the roles of the syncytin proteins, which are derived from Env proteins encoded in ERVs, in cell–cell fusion during placentation [12] and the roles of ERVs in establishing and/or maintaining the pluripotency of embryonic stem cells [13,14]. Regarding immunity, most studies suggested its enforcement by retroelements. ERV-derived peptides positively select developing thymocytes and enhance mature T-cell activation [15], suggesting their roles in adaptive immunity. Retroelements also activate innate immunity when they are transcribed or upon their reverse transcription into complementary DNA (cDNA) [16,17,18]. It was also demonstrated that retroelements help the host to acquire viral sequences, which may function as a novel type of immunity against related viruses [19]. In contrast to these reported immune-enhancing roles, we demonstrated a possible unique role of MLT-int RNA in suppressing the innate immune system in this study (Figure S4A).

At present, how MLT-int suppresses IFN induction remains to be addressed. Retroelements exert their functions as regulatory DNA, non-coding RNA (ncRNA), or protein. For example, members of the MER41 family contribute to the activation of immune-related genes as an enhancer sequence [20]. When some ERV-encoded proteins are expressed, they can suppress infection by related viruses in a dominant-negative manner [21]. The *MLT-int* element on chromosome 4 does not contain a coding region longer than 200 nt, suggesting that the element likely functions as an ncRNA. Consistently, MLT-int-containing transcripts were distributed mainly in the nucleus (Figure 3B), where most long ncRNAs play their roles. Because of their viral origin, nucleic acid replication intermediates of ERVs are reportedly recognized by host innate sensors of non-self nucleic acids [17,18]. However, we did not detect any MLT-int RNA in the immunoprecipitates with retinoic acid-inducible gene I (RIG-I), a major sensor of non-self RNAs (data not shown) [22]. Further investigation is required for a better understanding of immune-modulating mechanisms of MLT-int RNA.

Based on abovementioned lines of evidence, we speculate a model of the roles of MLT-int RNA in innate immune tuning (Figure S4A). Upon vaccination, immunogen induces the expression of IFN, which upregulates the expression of hundreds of ISGs and also MLT-int-containing transcripts. After elimination of immunogen at the post-immunization period, MLT-int RNA prompts termination of IFN signaling by suppressing IFN expression. Thus, this may be a mechanism to extinguish excessive innate immune activation, which is harmful to the host, once immune stimuli are removed. We analyzed additional RNA-seq data at 14 days after the low-dose vaccination (GSE98212) to reveal the expression change of ISGs in the later phase of vaccination. Among previously annotated core ISGs [23], we chose antigen-presentation-related genes to be analyzed because these ISGs are most likely related to vaccination. The expressions of most of these ISGs at 14 days after vaccination in the “High MLT-int” group were below those before vaccination, while those at 14 days after vaccination in the “Low MLT-int” group were still higher than those before vaccination (Figure S4B). Although this is consistent with our speculated model, we cannot conclude at this time that MLT-int-containing transcripts contribute to the suppression of immune responses in the later phase of vaccination, because ISG induction can be affected by multiple factors. Further evaluation of this model and its biological relevance is clearly required in the future. The unique subcellular localization and immune-suppressing property of the MLT-int element will provide novel insight into retroelement functions and also a novel target to modulate host immune systems. Because we only focused on the function of MLT-int-containing transcripts in immune tuning in this study, further mining of the roles of retroelements in other processes will accelerate our understanding of the fine regulation of various biological processes and facilitate the development of novel interventions to control them.

## 4. Materials and Methods

### 4.1. RNA-seq Analysis

The expression data used in this study are publicly available and described in a previous study [6]. The following accessions in GSE98212 were used: high-dose vaccination (GSM2589377, GSM2589378, GSM2589379, GSM2589380, GSM2589381, GSM2589382, GSM2589383, GSM2589384, GSM2589385, GSM2589386, GSM2589389, GSM2589390, GSM2589393, GSM2589394, GSM2589397, GSM2589398, GSM2589401, GSM2589402, GSM2589405, and GSM2589406) and low-dose vaccination (GSM2589407, GSM2589409, GSM2589410, GSM2589411, GSM2589413, GSM2589414, GSM2589415, GSM2589417, GSM2589418, GSM2589419, GSM2589421, GSM2589422, GSM2589423, GSM2589425, and GSM2589426). Approximately 36–50 million high-quality 100-bp paired-end reads per sample were mapped to the human genome (GRCh38) using Hisat2 (version 2.1.0) with default settings [24]. After Hisat2, we extracted the first primary alignment for every read with samtools command (view -F256) to remove multi-hit alignments. Repeat elements were downloaded from the University of California at Santa Cruz (UCSC) RepeatMasker track (GRCh38). We computed the median length and milliDiv (base mismatches in parts per thousand) for each repeat family (MaLR, 338 bp (threshold of the median length), 211 (threshold of milliDiv value); ERV1, 381 bp, 163; ERVK, 660 bp, 86; ERVL, 291 bp, 247; LINE, 283 bp, 216; and SINE, 296 bp, 119), and collected retroelements with a length longer than the median and milliDiv below the median value for expression analysis. Differential expression levels of retroelements were computed in a strand-specific manner using BEDTools with a minimum of 1-bp overlap (http://bedtools.googlecode.com), and the expression of sense-strands was analyzed except for Figure S1C. Reads per kilobase of exon per million mapped reads (RPKM) of a retroelement were calculated as follows: the total number of reads mapped on all loci of one element was divided by the total length (kilobase) of all loci of the element in the human genome, then normalized by million mapped reads. For the analysis of the composition of retroelement-containing transcripts (Figure S1A,B), the sum of RPKM of elements in a family was used for a representative value of the family. 

For differential gene expression analysis, the GRCh38.88 Ensembl gtf file was downloaded from Ensembl (http://asia.ensembl.org) and applied as a gene annotation file. The low-dose vaccination sample data were mapped to the human genome (GRCh38) using Tophat2 (version 2.0.13) with parameter (-g 1), and the Cufflinks algorithm (version 2.2.1) was used to calculate gene expression value (fragments per kilobase per million mapped reads: FPKM) with the Ensembl gtf file [25]. For comparison of ratios of gene expressions at 14 days after vaccination to those before vaccination (“Post 14 d/Pre” ratios) among low-dose vaccination subjects (Figure S4B), cluster analysis was done based on Ward’s method and square Euclidean distance. We used the average of expression of MLT-int-containing transcripts before, at 24 h after, and at 14 days after vaccination as representative MLT-int expression values of each subject. The average of these representative MLT-int expression values was used as a threshold for categorizing the “High MLT-int” and “Low MLT-int” groups.

### 4.2. Plasmids

The plasmid encoding the *MLT-int* element, pCMV-MLT-int, was generated by subcloning the full length of the *MLT-int* element (chr4:88,496,844–88,498,576 of GRCh38), which included the accompanied upstream *MLT1A0* sequence (chr4: 88,496,844–88,497,157 of GRCh38) in addition to the *MLT-int* sequence (chr4:88,497,185–88,498,576 of GRCh38), into pCMV-HA (Takara, Otsu, Japan). The plasmid for evaluating the *MLT1A0* promoter activity, pGLuc-MLT1A0, was generated by subcloning the *MLT1A0* sequence into pGLuc-Basic (New England Biolabs, Ipswich, MA, USA). The reporter plasmid for the IFN promoter assay, p125-Luc, was kindly provided by Prof. Fujita (Kyoto University) [26].

### 4.3. Primers

The sequences of the primers used in this study were as follows:

MLT-int-forward primer (OU223), 5′–GGA GAA TCA AAA GCA CCC AA–3′;

MLT-int-reverse primer (OU224), 5′–AAC TCA AAA TGG CAT GCA AC–3′;

*HERC5* forward primer (OU424), 5′–CGA ACT CTT GCA CCG TCT CA–3′;

*HERC5* reverse primer (OU425), 5′–CCT CAA TTG CTG CCG ACC TA–3′;

*GAPDH* forward primer [27,28], 5′–AGC GAG ATC CCT CCA AAA TC–3′;

*GAPDH* reverse primer [27,28], 5′–AAA TGA GCC CCA GCC TTC TC–3′.

### 4.4. Cells

The 293T cells (a human embryonic kidney cell line) were cultured in Dulbecco’s modified Eagle’s medium (DMEM) supplemented with 5% fetal bovine serum (FBS).

### 4.5. Subcellular Fractionation

Subcellular fractionation was conducted as described previously [29]. Briefly, 293T cells were suspended with buffer A (10 mM HEPES pH 7.9, 1.5 mM MgCl_2_, 10 mM KCl, 0.5 mM DTT) and incubated on ice for 10 min. After centrifugation at 300× *g*, the cells were re-suspended with buffer A containing 0.05% NP-40. The supernatant and the pellet were harvested as a cytoplasmic and a nuclear fraction, respectively, by centrifugation at 300× *g* for 10 min.

### 4.6. Luciferase Reporter Assay

For the *MLT1A0* promoter assay, 1 × 10^5^ 293T cells were seeded in 24-well plates. After the one-day incubation, the cells were transfected with 0.2 µg of pGLuc-Basic or pGLuc-MLT1A0 together with 10 ng of pCMV-CLuc (New England BioLabs) using Lipofectamine 2000 (Invitrogen, Gaithersburg, MD, USA). At 24 h after the transfection, the cells were further transfected with 0.4 µg of polyI:C (Sigma-Aldrich, St. Louis, MO, USA). At 48 h after reporter transfection, the luciferase activity of the cells was measured using the *Gaussia* and *Cypridina* Luciferase Assay Kit (New England BioLabs) according to the manufacturer’s instructions. For the IFN promoter assay, 293T cells were transfected with 0.2 µg of p125-Luc and 10 ng of pRL-TK (Promega, Fitchburg, WI, USA) with or without 0.2 µg of pCMV-MLT-int. At 24 h after reporter transfection, the cells were further transfected with 0.4 µg of polyI:C. For the ISRE reporter assay, 293T cells were transfected with 0.2 µg of pISRE-Luc (Agilent Technology, Wilmington, DE, USA) and 10 ng of pRL-TK with or without 0.2 µg of pCMV-MLT-int. At 24 h after reporter transfection, the cells were further treated with 1 KU of universal IFN (Sigma-Aldrich). At 48 h after reporter transfection, the luciferase activity of the cells was measured using the Dual-Luciferase Reporter Assay System (Promega) according to the manufacturer’s instructions.

### 4.7. Real-Time PCR

Total RNA was extracted from infected cells and reverse-transcribed using a Verso cDNA Synthesis Kit (Thermo Scientific, Waltham, MA, USA) with a random hexamer or oligo dT as a primer. Quantitative real-time PCR (qRT-PCR) assays were carried out using gene-specific primers with a QuantStudio 6 real-time PCR system (Thermo Fisher Scientific) according to the manufacturer’s instructions. PCR reactions were incubated at 95 °C for 20 s, followed by 40 amplification cycles of annealing and extension at 60 °C for 20 s and denaturation at 95 °C for 1 s. The amount of GAPDH mRNA was used to standardize the total amount of cDNA. For subcellular fractionation, RNA in a fraction was extracted from the equal proportion of each fraction. 

### 4.8. Statistics

Statistical significance was assessed using a two-tailed Student’s *t*-test with a threshold of *p* < 0.05.

## Supplementary Materials

Supplementary materials can be found at https://www.mdpi.com/1422-0067/20/12/2875/s1.

## Abbreviations

ERV	endogenous retrovirus
IFN	interferon
ISG	IFN-stimulated gene
ISRE	IFN-stimulated response element
LINE	long interspersed element
MaLR	mammalian apparent LTR-retrotransposon
ncRNA	non-coding RNA
RIG-I	retinoic acid-inducible gene I
SINE	short interspersed element
