# Supplementary material for: Identification of a Retroelement-Containing Human Transcript Induced in the Nucleus by Vaccination
Source: Int J Mol Sci. 2019 Jun 13;20(12):2875. doi: 10.3390/ijms20122875 (PMC6627062; doi:10.3390/ijms20122875)
Supplement: Supplementary file 1 [file ijms-20-02875-s001.pdf]

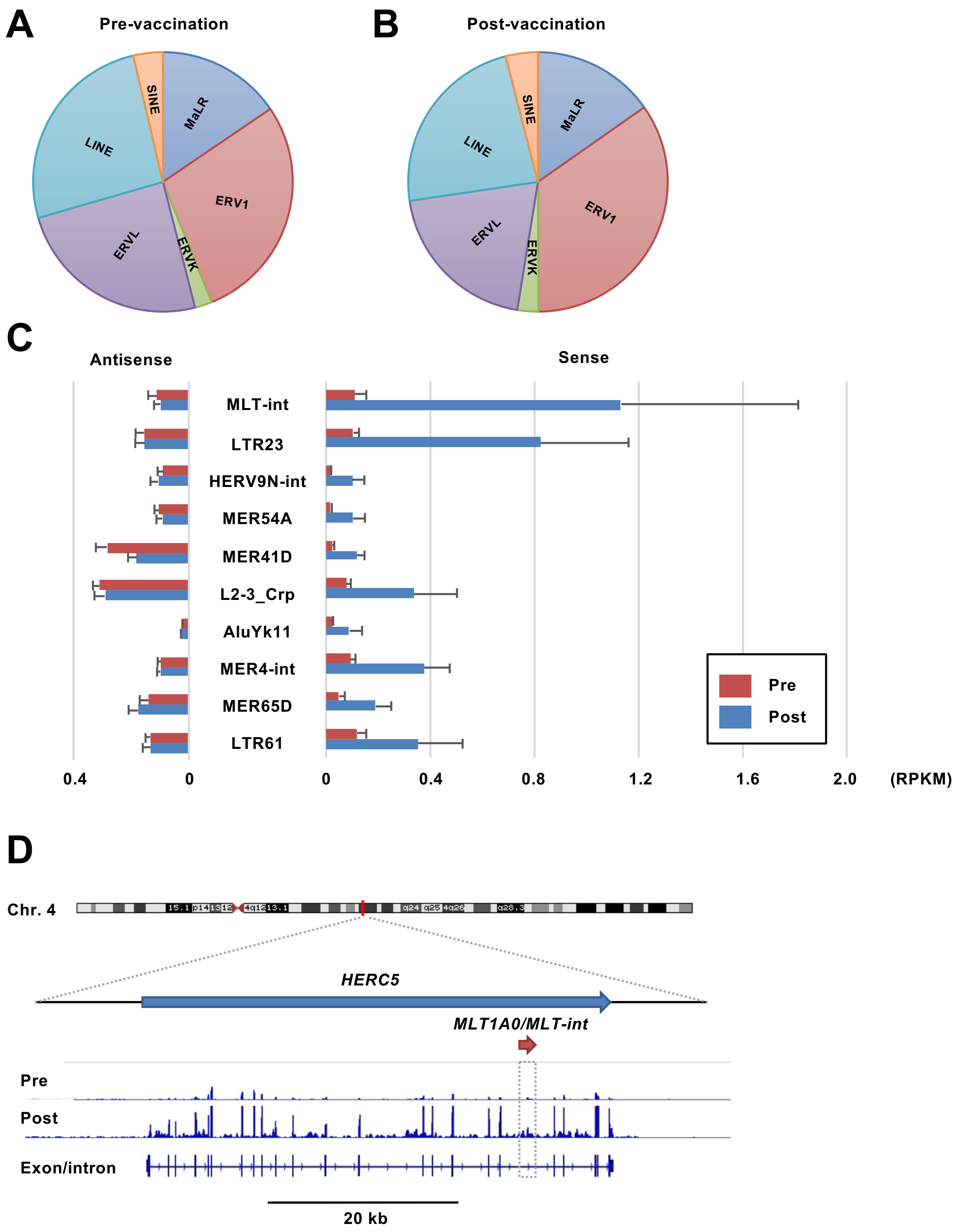

Supplemental Figure S1. Expression profiling of transcripts containing retroelements during vaccination at a high dose.

(A, B) Diversity of transcripts containing retroelements expressed in peripheral blood cells before (A) and at 24 h after (B) vaccination at a high dose (GSE98212, high-dose, N = 10). (C) Top 10 retroelements in upregulated transcripts during vaccination. (D) The *MLT-int* locus within the *HERC5* gene on chromosome 4. Representative patterns of mapped reads on the locus at pre- and post-vaccination is shown.

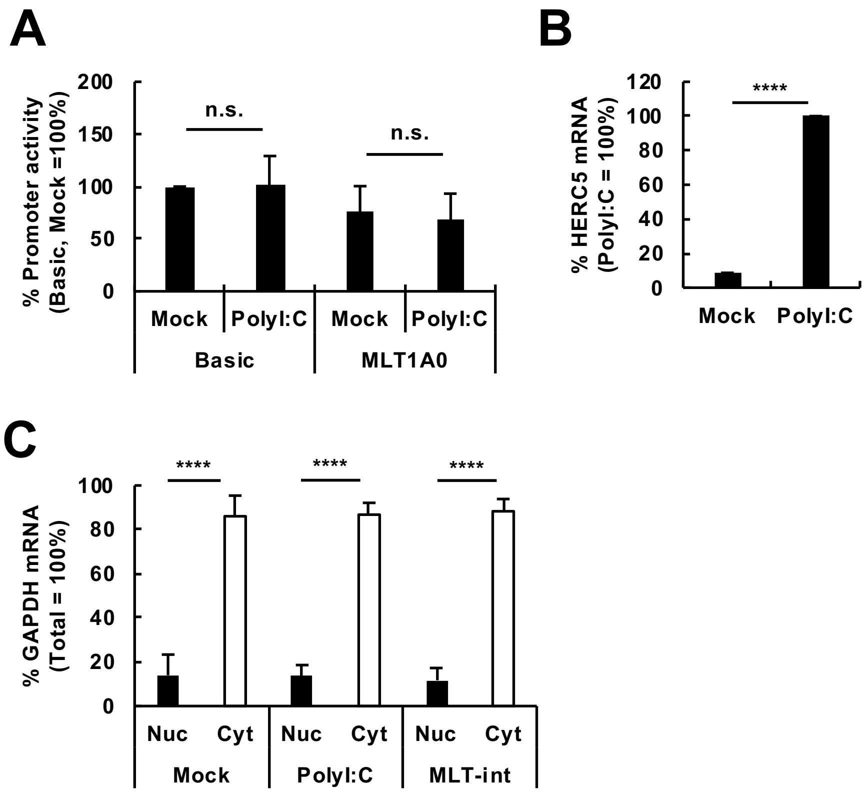

Supplemental Figure S2. Evaluation of properties of the MLT-int element.
(A) The effect of polyI:C treatment on the MLT1A0 promoter. 293T cells were transfected with pGLuc-Basic (Basic) or pGLuc-MLT1A0 (MLT1A0), together with pCMV-CLuc. After 24 h of incubation, the cells were transfected with polyI:C and further incubated for 24 h. (B) Induction of HERC5 mRNA by polyI:C treatment. (C) Subcellular localization of the GAPDH mRNA in Mock, polyI:C-treated and MLT-int-expressing 293T cells. The values are expressed as the mean + S.E. of at least three independent experiments. ****, *P* < 0.001; n.s., no significance.

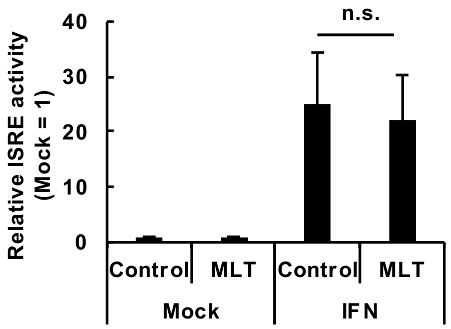

Supplemental Figure S3. ISRE activity with or without MLT-int RNA.
293T cells were transfected with a plasmid expressing MLT-int RNA. After 24 h of incubation, the cells were treated with 1 KU of IFN and further incubated for 24 h. The values are expressed as the mean + S.E. of at least three independent experiments. n.s., no significance.

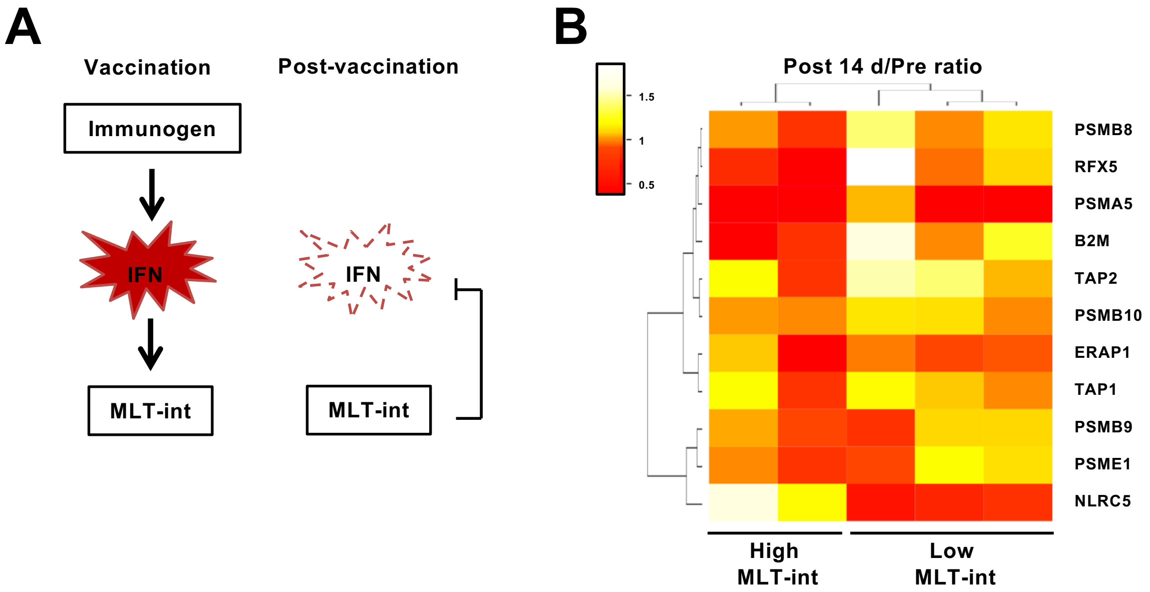

Supplemental Figure S4. Expression profiling of retroelements during vaccination. (A) A proposed model for the function of MLT-int. During vaccination (Vaccination), immunogen upregulates IFN, which induces MLT-int expression. Once immunogen is eliminated (Post-vaccination), MLT-int accelerates termination of IFN expression. (B) Heatmap of the Post 14 d/Pre expression ratios of antigen-presentation-related genes annotated as core ISGs (see Shaw, et al., 2017). Each column represents a distinct subject, while each row represents an antigen-presentation-related gene. Subjects with MLT-int expression above the average were categorized into the “High MLT-int” group, whereas those below the average were categorized into the “Low MLT-int” group.
